# Inter-assay precision of clonogenic assays for radiosensitivity in cancer cell line A549

**DOI:** 10.18632/oncotarget.24448

**Published:** 2018-02-07

**Authors:** Endang Nuryadi, Tiara Bunga Mayang Permata, Shuichiro Komatsu, Takahiro Oike, Takashi Nakano

**Affiliations:** ^1^ Department of Radiation Oncology, Gunma University Graduate School of Medicine, Gunma, Japan; ^2^ Department of Radiotherapy, Dr. Cipto Mangunkusumo National General Hospital, Jakarta, Indonesia

**Keywords:** clonogenic assay, radiosensitivity, cancer cell, meta-analysis, precision medicine

## Abstract

Clonogenic assays are the gold standard for determining radiosensitivity, which governs tumor response to radiation therapy. Although multiple studies of clonogenic assays on cancer cell lines have been published, the robustness of this technique has not been examined by comparative analysis of data from different studies. To address this issue, we investigated the inter-assay precision of clonogenic assays by analyzing in-house and published data on A549, a cell line frequently studied in this context. The coefficients of variation for SF2, the surviving fraction after 2 Gy irradiation, and D10, the radiation dose that reduces survival to 10%, were below 30% for both in-house data obtained from 20 independent experiments performed under consistent experimental settings (i.e., radiation type, dose rate, and timing of cell seeding) and data collected from 192 publications using diverse experimental settings. Multivariate analyses of the published data revealed that timing of cell seeding significantly affected SF2. These data indicate that SF2 and D10 of clonogenic assay have acceptable inter-assay precision, and that timing of cell seeding influences the inter-assay precision of SF2. These results provide a rationale for combined analysis of published clonogenic assay data, which may help to discover robust biological properties associated with tumor radiosensitivity.

## INTRODUCTION

Clonogenic assays are the gold standard for determining reproductive cell death induced by ionizing radiation (IR) [1]. Multiple studies show that cellular radiosensitivity, as determined by clonogenic assay in cancer cell lines, is relevant to the tumor response to radiation therapy [2]. On the other hand, recent advances in computer science, including machine learning, and in omics technologies, such as next-generation sequencing, enable us to conduct high-throughput analysis of big data with the goal of discovering biomarkers and therapeutic targets useful for precision cancer medicine [3]. Multiple data resources, such as the Cancer Cell Line Encyclopedia (CCLE) and Catalogue of Somatic Mutations in Cancer [4, 5], provide extensive omics information about cancer cell lines, including mutation and mRNA expression profiles. Similarly, an enormous number of publications report radiosensitivity of cancer cell lines as assessed by clonogenic assays. Bioinformatics analyses using the available clonogenic assay and omics data in combination have significant potential for the discovery of biological properties associated with radiosensitivity, which will be useful for prediction of tumor responses to radiation therapy. However, the robustness of clonogenic assays has not been examined by comparative analysis of data from different studies.

To address this issue, we evaluated the inter-assay precision of clonogenic assay by analyzing in-house and published data on A549, a cell line frequently studied using this method. Using in-house data acquired from 20 independent *in vitro* experiments and data acquired from 192 publications identified by a comprehensive literature search, we analyzed the coefficient of variation (CV) of clonogenic survival and the influence of experimental settings on assay results.

## RESULTS

### Data acquisition from the literature

**Figure 1:**
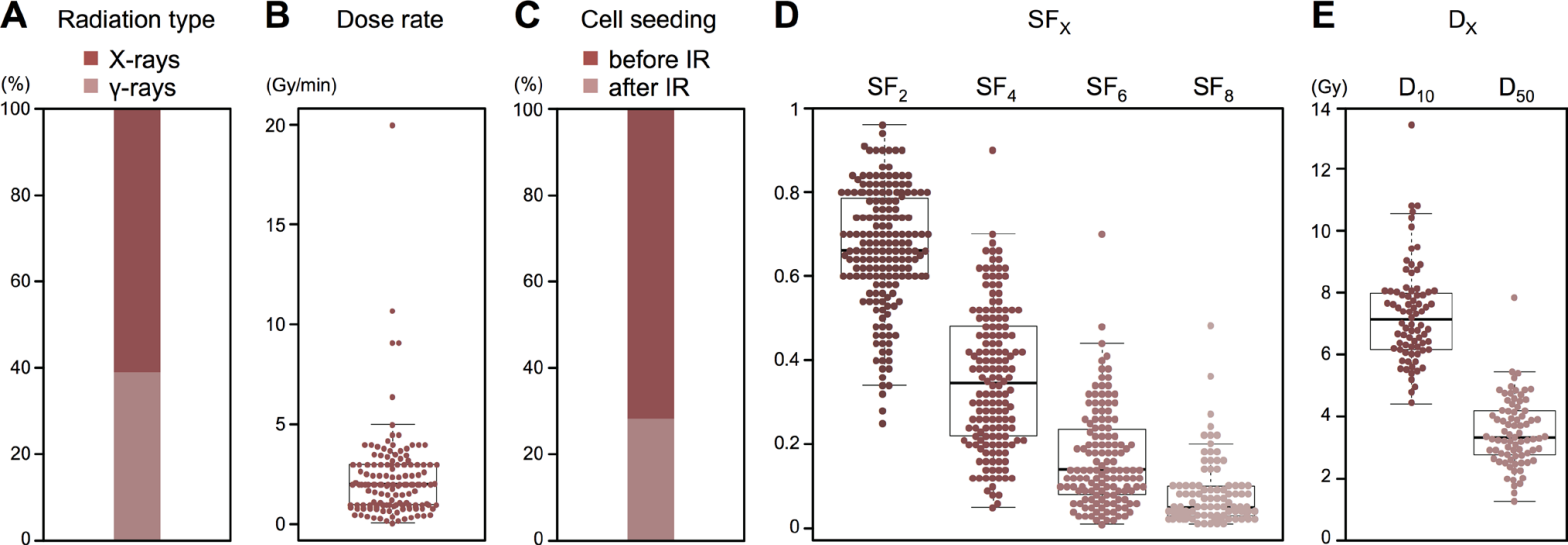
Summary of clonogenic assay data acquired from publications (**A**) Radiation type (*n* = 188). (**B**) Dose rate (*n* = 144). (**C**) Timing of cell seeding (*n* = 192). (**D**) SF_2_ (*n* = 179), SF_4_ (*n* = 156), SF_6_ (*n* = 136), and SF_8_ (*n* = 92). (**E**) D_10_ (*n* = 79) and D_50_ (*n* = 79).

To analyze inter-assay precision of clonogenic assays, we first conducted a literature search to compile data obtained using these methods. We sought to acquire the greatest quantity of data on clonogenic assays from a specific human cancer cell line treated with X-rays or γ-rays. To this end, we performed a comprehensive literature search for 1039 human cancer cell lines registered in CCLE (see *Literature search* in MATERIALS AND METHODS for details). We found that the lung cancer cell line A549 is most frequently used in studies of sensitivity to X-rays or **γ**-rays using clonogenic assays, and has been described in 192 papers. From these papers, we extracted the values of SF_2_, SF_4_, SF_6_, SF_8_, D_10_, and D_50_ as endpoints for clonogenic survival (see *Acquisition of clonogenic survival data from the literature* in MATERIALS AND METHODS for details). In addition, we extracted information regarding experimental settings that can affect clonogenic survival, including radiation type, dose rate, and timing of cell seeding [1]. The contents of the dataset are summarized in Figure 1. The entire dataset is described in Supplementary Table 1.

### Data acquisition from *in vitro* experiments

**Figure 2:**
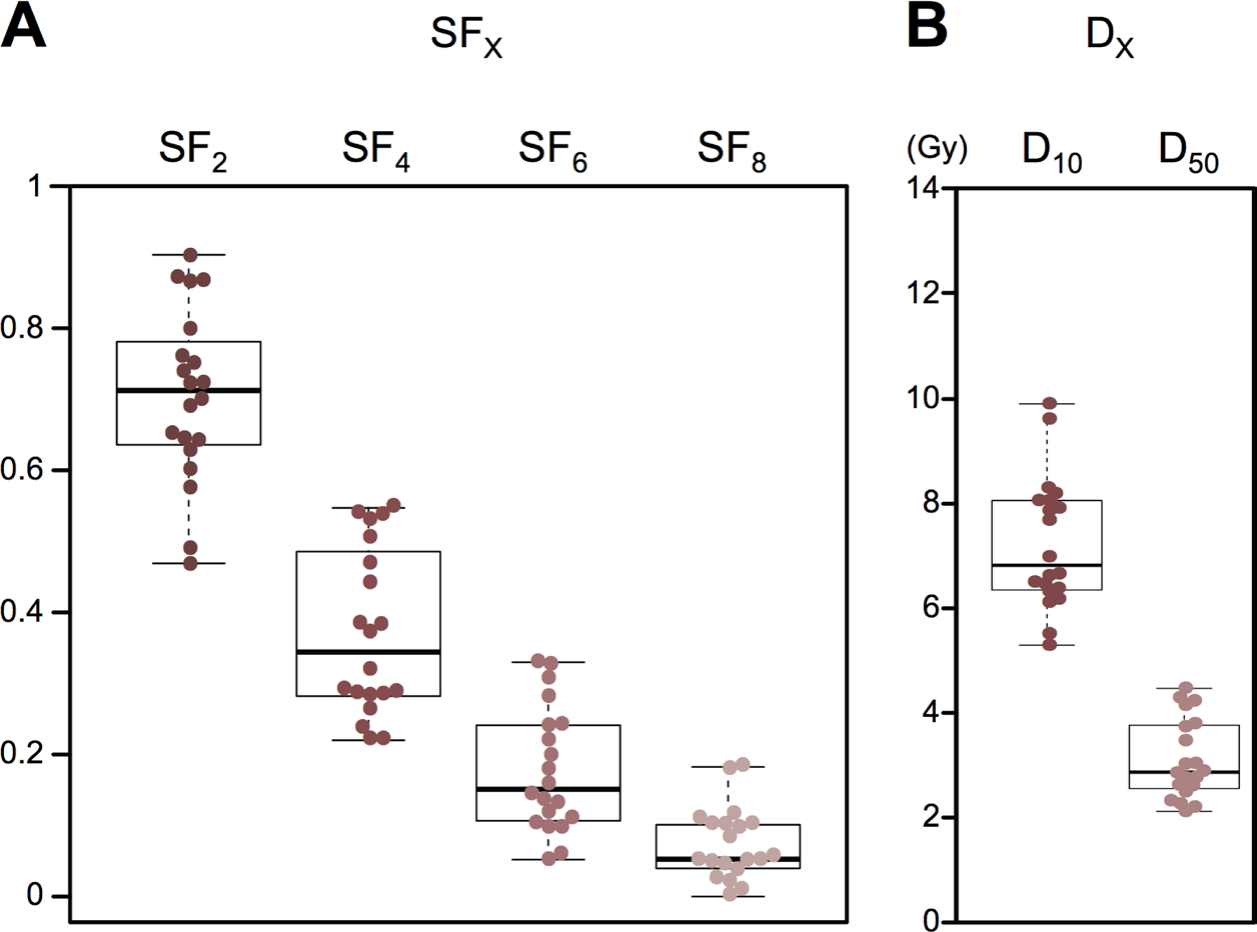
Summary of clonogenic survival data acquired from *in vitro* experiments **(A)** SF_2_, SF_4_, SF_6_, and SF_8_ (*n* = 20). **(B)** D_10_ and D_50_ (*n* = 20).

Next, we acquired in-house data from clonogenic assays on IR-treated A549 cells. We performed 20 independent experiments using consistent experimental settings (radiation type: X-rays, dose rate: 1.14 Gy/min, and timing of cell seeding: before irradiation), and acquired the values of SF_2_, SF_4_, SF_6_, SF_8_, D_10_, and D_50_ (Figure 2, Supplementary Table 2).

### Analysis of inter-assay precision for clonogenic survival endpoints

**Figure 3:**
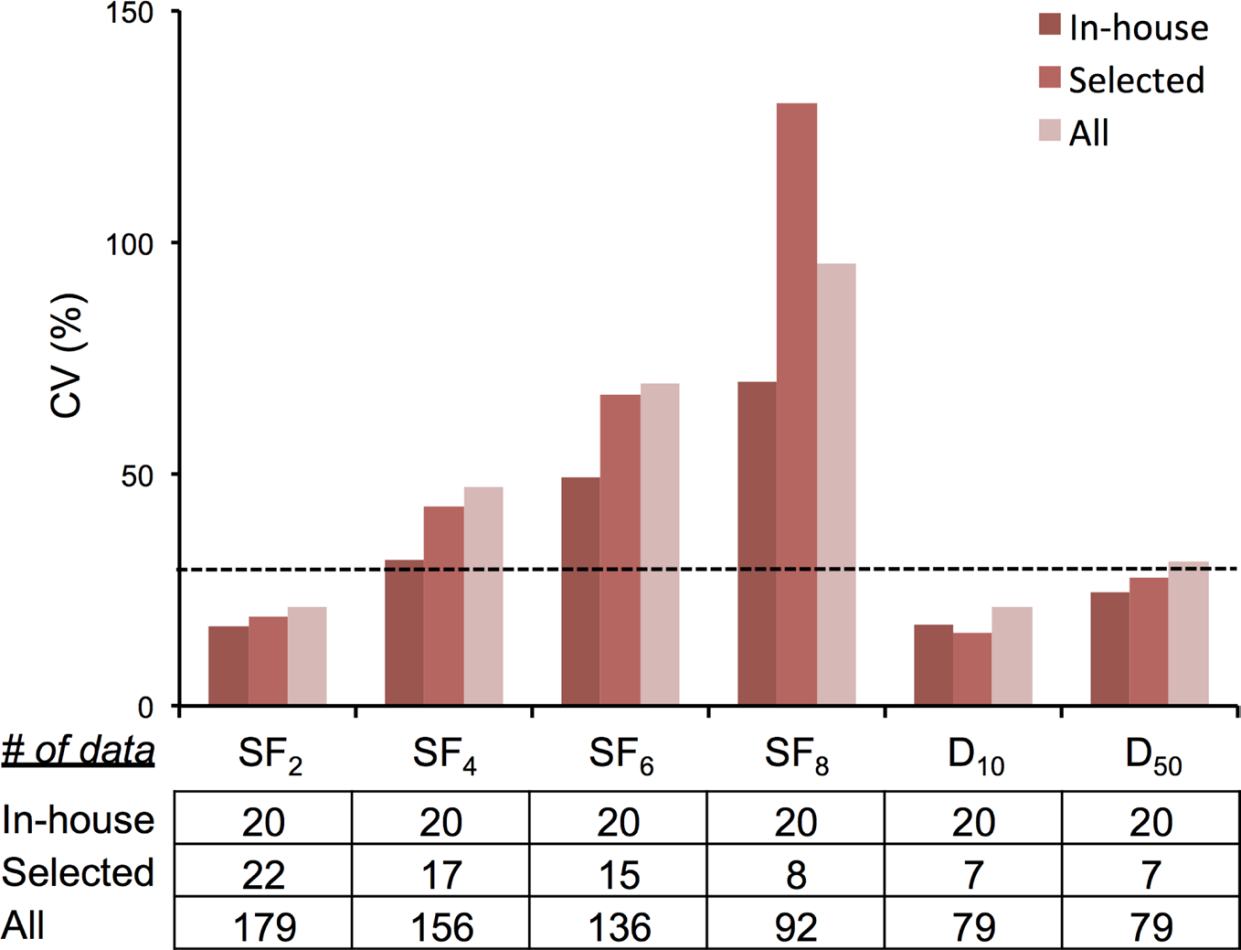
CV values of clonogenic survival In-house, data from in-house experiments. Selected, data from selected publications that employed the following experimental settings: radiation type: X-rays; dose rate: 1.14 ± 0.5 Gy/min; and timing of cell seeding: before irradiation. All, data from all publications. Dotted line indicates CV of 30%.

To investigate the inter-assay precision of clonogenic assays, we examined the CV values of the clonogenic survival endpoints, following the recommendation from the Food and Drug Administration (FDA) that a CV of lower than 30% is acceptable for assessment of potential biomarkers [6, 7]. For the in-house data obtained using consistent experimental settings, the CV values were below 30% for SF_2_, D_10_, and D_50_ (Figure 3). For published data obtained using experimental settings comparable to those of our in-house experiments (i.e., radiation type: X-rays, dose rate: 1.14 ± 0.5 Gy/min, timing of cell seeding: before irradiation), CV values of clonogenic survival endpoints were also below 30% for SF_2_, D_10_, and D_50_ (Figure 3), validating the analysis of the in-house experiments. These data indicate that the clonogenic assays have acceptable inter-assay precision as a measure of radiosensitivity when experimental settings are consistent, and when SF_2_, D_10_, and D_50_ are used as endpoints.

To investigate whether clonogenic assays have acceptable inter-assay precision under diverse experimental settings, as is the case for data obtained by different laboratories and clinics, we examined CV values of clonogenic survival endpoints for all publications without restricting the experimental settings. Interestingly, CV values of SF_2_ and D_10_ remained below 30%, comparable to those of the in-house experiments (Figure 3). Taken together, these data indicate that clonogenic assays have acceptable inter-assay precision as a measure for radiosensitivity, regardless of experimental settings related to radiation type, dose rate, and timing of cell seeding, when SF_2_ and D_10_ are used as endpoints.

Finally, we investigated the influence of differences in experimental settings on clonogenic survival, using all the published data. Univariate analysis revealed that radiation type (X-rays or γ-rays), dose rate, and timing of cell seeding (before or after irradiation) did not have a significant impact on SF_2_, SF_4_, SF_6_, SF_8_, D_10_, or D_50_ (Table 1), although we did observe a trend toward higher SF_2_ values for cells seeded before irradiation compared with those seeded after irradiation (*P* = 0.06). Consistent with this, multivariate analysis revealed that the timing of cell seeding had a significant impact on SF_2_ (*P* = 0.04), but we observed no significant impact of these experimental settings on any other clonogenic survival endpoints tested (Table 2). These data suggest that the timing of cell seeding influences SF_2_.

**Table 1:** Univariate analysis of the influence of experimental settings on clonogenic survival

Parameter	SF_2_	SF_4_	SF_6_	SF_8_	D_10_	D_50_
Radiation type^*^	0.29	0.29	0.29	0.94	0.74	0.85
Dose rate	0.76	0.80	0.62	0.85	0.69	0.66
Timing for cell seeding^**^	0.06	0.34	0.23	0.23	0.59	0.99

The differences in SF_2_, SF_4_, SF_6_, SF_8_, D_10_, or D_50_ between different radiation types and between timings of cell seeding were examined by Mann–Whitney *U* test. The correlation of dose rate with SF_2_, SF_4_, SF_6_, SF_8_, D_10_, or D_50_ was examined by Spearman’s rank correlation test. *P* values are shown. *X-rays vs. γ-rays; **before irradiation vs. after irradiation.

**Table 2:** Multivariate analysis of the influence of experimental settings on clonogenic survival

Parameter	SF_2_	SF_4_	SF_6_	SF_8_	D_10_	D_50_
Radiation type^*^	0.63	0.49	0.57	0.47	0.95	0.34
Dose rate	0.50	0.24	0.69	0.53	0.95	0.77
Timing for cell seeding^**^	0.04	0.63	0.47	0.24	0.83	0.91

The influence of radiation type, dose rate, and timing of cell seeding on SF_2_, SF_4_, SF_6_, SF_8_, D_10_, or D_50_ was examined by multiple linear regression. *P* values are shown. *X-rays vs. γ-rays; **before irradiation vs. after irradiation.

## DISCUSSION

In this study, we investigated inter-assay precision of clonogenic assays as a measure for radiosensitivity, using in-house and published data on A549, a cell line frequently studied using this method. The CV values of SF_2_ and D_10_ were below 30% for both datasets regardless of experimental settings, indicating that clonogenic assay has acceptable inter-assay precision as a biomarker for tumor radiosensitivity [6, 7]. To the best of our knowledge, this is the first study to examine inter-assay precision of clonogenic assay using a meta-analytic approach. In addition, considering the comprehensive nature of our literature search, the scale of the compiled data represents the maximum amount of available data regarding clonogenic assay of a specific cell line.

To elucidate the biological properties associated with tumor responses to radiation therapy, several groups analyzed the association of radiosensitivity, which they assessed by performing clonogenic assays, with omics data. However, due to the time- and labor-intensiveness of the assay and the cost of purchasing cell lines, the number of cell lines analyzed in a given study has been limited, below a hundred in most cases [8, 9]. The results of this study indicate acceptable inter-assay precision for SF_2_ and D_10_ in clonogenic assay, providing a rationale for combined analysis of multiple published datasets, leading to greater statistical power. Comprehensive correlation analysis using published clonogenic assay data with available omics data, such as gene mutation status and mRNA expression profiles, will contribute to elucidation of robust biological properties associated with tumor radiosensitivity, which will in turn promote the development of precision cancer medicine for radiation therapy.

The influence of experimental settings on radiosensitivity has been extensively investigated over several decades, with various endpoints including the responses of human tissues, animals, and cultured cells [10–12]. However, little solid evidence is available from clonogenic survival assays of human cancer cell lines. The results of this study revealed that radiation type (X-rays or γ-rays) and dose rate do not significantly impact SF_2_ and D_10_ which exhibit acceptable inter-assay precision (i.e., CV <30%) under consistent experimental settings. Although these results do not exclude the possibility that specific treatment settings affect clonogenic survival, we can at least conclude that the inter-assay precision of SF_2_ and D_10_ obtained using diverse radiation types and dose rates is comparable to that obtained using consistent treatment settings when the data are subjected to meta-analysis, as in this study. The results of this study also revealed that the timing of cell seeding significantly influences SF_2_. Therefore, theoretically, the data should be stratified by the timing of cell seeding when SF_2_ values are subjected to meta-analysis. On the other hand, the CV values for “before irradiation” and “after irradiation” dataset is 19% and 24%, respectively, which is not evidently smaller than that for the entire dataset (21% as shown in Figure 3), indicating that the stratification by the timing of cell seeding is not necessarily beneficial for the improvement of data precision. In addition, from a practical standpoint, the number of papers reporting clonogenic assay results for a given cell line is not always high as that for A549 (Supplementary Figure 1); in such cell lines, stratification by the timing of cell seeding may decrease the amount of data sets available for analysis, which lowers the reliability of the results. Therefore, the stratification by the timing of cell seeding for the meta-analysis of SF_2_ may be employed case-by-case considering these things together.

SF_2_, an endpoint that has been utilized for radiosensitivity assessment in the clinic more commonly than SF_4_, SF_6_, and SF_8_, had the lowest CV values [2, 10]. These data shed light on the validity of the traditional use of SF_2_ for prediction of clinical radiosensitivity, although the mechanisms underlying the smaller CV for SF_2_ remain to be elucidated. On the other hand, it is reasonable that CV values for D_10_ and D_50_ calculated using surviving fractions at multiple dose points were generally lower than those for the surviving fractions at each dose point. Together, these findings indicate that, among the endpoints for clonogenic assays tested in this study, SF_2_ is the best in terms of both inter-assay precision and convenience of data acquisition.

It may be worthy to note that 85% (6/7) of the papers that provided high outliers employed “before irradiation” as the timing of cell seeding, while 100% (3/3) of the papers that provided low outliers employed “after irradiation” (Figure 1D, 1E and Supplementary Table 1); any other specific experimental settings were commonly employed in those papers. Although univariate and multivariate analyses in the present study did not show significant influence of the timing of cell seeding on the survival endpoints other than SF_2_, this point should be further pursued in future.

This study has the following limitations. First, no firm consensus has been established regarding the criteria for inter-assay precision for assessment of potential biomarkers. Although we followed the recommendation of the FDA [6, 7], inter-assay precision of clonogenic assay should be further investigated in the future, taking into account other criteria. Secondly, in the literature search, we excluded papers in which cells were irradiated as suspensions, because these represented a small minority of studies (2 of 194 papers reporting clonogenic assay of IR-treated A549 cells). Also, due to the low statistical power, we were not able to analyze the influence of radiation source (e.g., ^60^Co, ^137^Cs, etc.) on clonogenic survival in greater detail. These factors can affect clonogenic survival, and should therefore be investigated in the future. Finally, we analyzed only one cell line; because the results may differ in other cell lines with genetic profiles distinct from those of A549 cells, an analysis using different cell lines should be performed in the future.

In summary, we conducted a comprehensive meta-analysis of in-house and published data on clonogenic assay for IR-treated A549 cells. The results showed that SF_2_ and D_10_ have acceptable inter-assay precision as endpoints for clonogenic survival. These results provide a rationale for the combined analysis of published clonogenic assay data, which may contribute to improvement of precision cancer medicine in radiation therapy by facilitating the discovery of robust biological profiles associated with tumor radiosensitivity.

## MATERIALS AND METHODS

### Literature search

All published data regarding 1039 human cancer cell lines registered in CCLE, treated with X-rays or γ-rays, were searched using PubMed in April to June of 2016 (Supplementary Figure 1). For each cell line listed in Supplementary Table 3, the search was conducted using the terms “*cell line name* AND (X-rays OR gamma rays OR radiation)”; both *Primary name* and *Aliases* described in Supplementary Table 3 were used for cell line *name*. Publications in languages other than English were excluded. Among the cell lines examined, the three with the most related papers were MCF7, A549, and HepG2 (1509, 1455 and 764 papers, respectively). Two radiation oncologists (EN and TBMP) examined all the manuscripts related to these three cell lines in their entirety, and identified publications containing data obtained from clonogenic assays after treatment with X-rays or γ-rays alone. Papers in which cells were treated with empty vector, siRNA, or reagent vehicles in the setting described as “radiation alone” were excluded because such treatments could potentially influence clonogenic survival. However, papers in which cells were treated with dimethyl sulfoxide (DMSO) in the setting described as “radiation alone” were not excluded, based on the observation that DMSO treatment does not influence clonogenic survival after irradiation (Supplementary Figure 2). Papers in which cells received irradiation in suspension were also excluded.

### Acquisition of clonogenic survival data from the literature

From 192 papers listed in Supplementary Table 1, the values for SF_2_, SF_4_, SF_6_, SF_8_, D_10_, and D_50_ were acquired as the endpoints for clonogenic survival. SF_X_ indicates the surviving fraction of cells irradiated with X Gy, whereas D_X_ indicates the radiation dose required for survival of X% [10]. For SF_2_, SF_4_, SF_6_, and SF_8_, the values were recorded if they were described in the manuscript or tables; if not, the values were determined by overlaying the electronic figures describing clonogenic survival on grid scales in semi-transparent display. The values were recorded up to two decimal places (Supplementary Figure 3). For the papers in which all values for SF_2_, SF_4_, SF_6_, and SF_8_ were available, D_10_ and D_50_ were calculated as previously described [13]. Briefly, survival curves were generated by fitting the surviving fraction to a linear-quadratic model: SF = exp(−αD −βD^2^), where SF is the surviving fraction and D is the dose. Then, the D_10_ and D_50_ values were calculated by solving the resulting equations for survivals of 50% and 10%, respectively.

### Cell culture

A549 cells were purchased from ATCC and cultured using DMEM (Gibco) supplemented with 10% fetal bovine serum (Sigma).

### Clonogenic assay

Clonogenic assay was performed as described previously [1, 13]. Briefly, cells were seeded on 6-well plates in specified numbers in triplicate; 12 hours later, the cells were exposed (or not) to X-ray irradiation (2, 4, 6, or 8 Gy) using a Faxitron RX-650 radiation source (100 kVp, 1.14 Gy/min; Faxitron Bioptics). After incubation for an additional 10 days, the cells were fixed with methanol and stained with crystal violet. Colonies of at least 50 cells were counted. The surviving fractions at 2, 4, 6, and 8 Gy were normalized to the corresponding unirradiated controls, yielding SF_2_, SF_4_, SF_6_, SF_8_, respectively. The values for D_10_ and D_50_ were acquired as described in *Acquisition of clonogenic survival data from the literature*, above. The average values for each clonogenic survival endpoint were used for subsequent analyses.

### Statistical analysis

Radiation type (i.e., X-rays and γ-rays) and the timing of cell seeding (i.e., before or after irradiation) were analyzed as categorical variables. Dose rate, SF_2_, SF_4_, SF_6_, SF_8_, D_50_, and D_10_ were analyzed as continuous variables. The differences in SF_2_, SF_4_, SF_6_, SF_8_, D_10_, or D_50_ between different radiation types and between the timings of cell seeding were examined by Mann–Whitney *U* test. The correlation of dose rate with SF_2_, SF_4_, SF_6_, SF_8_, D_10_, or D_50_ was examined by Spearman’s rank correlation test. The influence of radiation type, dose rate, and the timing of cell seeding on SF_2_, SF_4_, SF_6_, SF_8_, D_10_, or D_50_ was examined by multivariate analysis using multiple linear regression. All statistical analyses were performed using EZR (Saitama Medical Center, Jichi University, Saitama, Japan), a graphical user interface for R (The R Foundation for Statistical Computing, Vienna, Austria, ver. 3.3.2) [14]. Differences were considered statistically significant at *P* values < 0.05.

## SUPPLEMENTARY MATERIALS FIGURES AND TABLES







## Abbreviations

CCLE: Cancer Cell Line Encyclopedia
CV: coefficient of variation
DMSO: dimethyl sulfoxide
FDA: Food and Drug Administration
IR: ionizing radiation
