# Supplementary material for: Inter-assay precision of clonogenic assays for radiosensitivity in cancer cell line A549
Source: Oncotarget. 2018 Feb 7;9(17):13706–12. doi: 10.18632/oncotarget.24448 (PMC5862609; doi:10.18632/oncotarget.24448)
Supplement: Supplementary file 1 [file oncotarget-09-13706-s001.pdf]

Inter-assay precision of clonogenic assays for radiosensitivity in cancer cell line A549

SUPPLEMENTARY MATERIALS

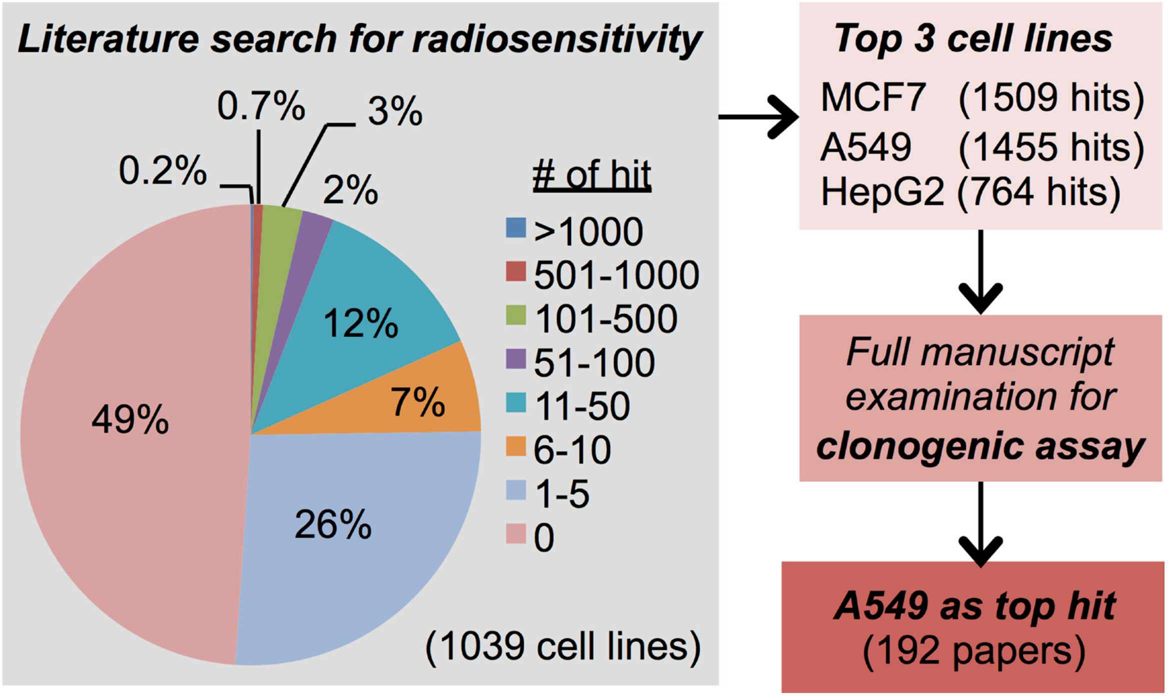

Supplementary Figure 1: Scheme for literature search.

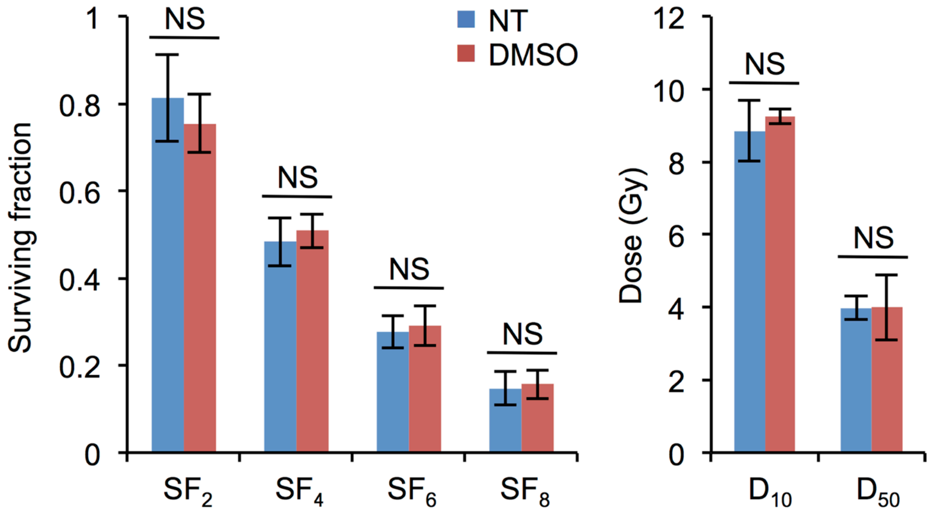

Supplementary Figure 2: Effect of DMSO on clonogenic survival of irradiated A549 cells. A549 cells treated with DMSO (Sigma) were irradiated with X-rays and subjected to clonogenic assay as described in MATERIALS AND METHODS ($n = 12$). The cells were exposed to 0.1% DMSO from 2 hours before irradiation until the time of colony staining. DMSO concentration was determined based on the maximal concentration described in publications collected from a literature search, in which DMSO concentration ranged from 0.001 to 0.1% (PMID: 9766667; 16928817; 16957930; 17875720; 22441439; 24366006; 24606853; 25692226). The differences in the clonogenic endpoints (i.e., SF2, SF4, SF6, SF8, D10, and D50) between non-treated (NT) and DMSO-treated cells were examined by non-paired two-tailed Student's *t*-test. Differences were considered statistically significant at P values < 0.05. NS, no statistically significant difference.

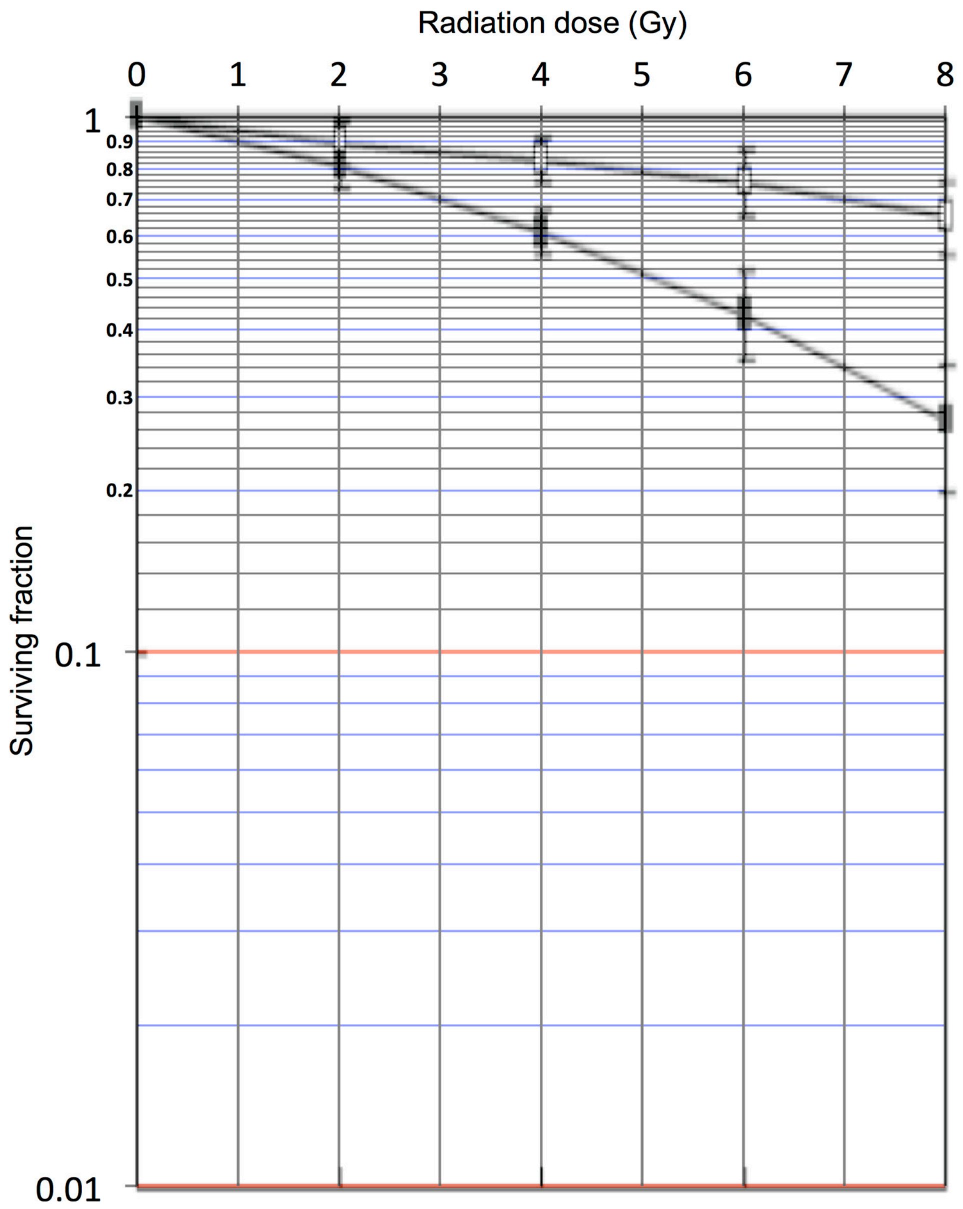

Supplementary Figure 3: Example of acquisition of clonogenic survival data from a published figure. A figure describing clonogenic survival is overlaid on grid scales in a semi-transparent display that enables measurement of the surviving fraction.

Supplementary Table 1: Clonogenic assay data for IR-treated A549 cells collected from the literatures. See Supplementary_Table_1

Supplementary Table 2: Clonogenic survival data for IR-treated A549 cells collected from *in vitro* experiments

Experiment	SF2	SF4	SF6	SF8	D10	D50
1	0.80	0.53	0.28	0.10	8.1	4.2
2	0.74	0.22	0.10	0.05	6.3	2.2
3	0.65	0.38	0.20	0.10	7.9	3.0
4	0.70	0.54	0.33	0.18	9.9	4.1
5	0.64	0.37	0.22	0.10	8.1	3.0
6	0.72	0.22	0.06	0.02	5.5	2.3
7	0.87	0.29	0.11	0.05	6.5	2.8
8	0.76	0.54	0.33	0.18	9.6	4.3
9	0.72	0.47	0.24	0.08	7.7	3.8
10	0.60	0.28	0.14	0.02	6.1	2.8
11	0.63	0.26	0.05	0.01	5.3	2.6
12	0.87	0.28	0.10	0.05	6.5	2.6
13	0.49	0.32	0.14	0.05	6.6	2.5
14	0.75	0.44	0.16	0.05	7.0	3.5
15	0.87	0.55	0.31	0.11	8.2	4.5
16	0.69	0.50	0.24	0.12	8.3	3.7
17	0.65	0.38	0.18	0.10	7.9	2.9
18	0.90	0.29	0.10	0.04	6.4	2.8
19	0.47	0.28	0.12	0.04	6.2	2.3
20	0.58	0.24	0.13	0.00	6.7	2.1

Supplementary Table 3: Cell line name for literature search. See Supplementary_Table_3
